# A case of persistent sciatic artery aneurysm with recurrent embolism

**DOI:** 10.1016/j.ijscr.2019.06.017

**Published:** 2019-06-13

**Authors:** Mutsuo Tanaka, Minoru Okamoto, Ken Okamoto, Toshihiro Fukui

**Affiliations:** a National Hospital Organization Kumamoto Medical Center, Department of Cardiovascular Surgery, 1-5 Ninomaru, Chuo-ku, Kumamoto-shi, Kumamoto Prefecture, 860-0008, Japan; b Kumamoto University Hospital, Department of Cardiovascular Surgery, 1-1-1 Honjo, Chuo-ku, Kumamoto-shi, Kumamoto Prefecture, 860-8556, Japan

**Keywords:** PSA, Persistent sciatic artery, PTA, Posterior-tibial artery, FA, Femoral artery, PA, popliteal artery, EVT, endovascular treatments, Persistent sciatic artery aneurysm, Limb ischemia, Peripheral vascular surgery

## Abstract

**Introduction:**

Persistent sciatic artery (PSA) is a rare congenital vascular anomaly. It has several clinical features with the formation of aneurysms. Among the complications, leg ischemia is severe problem and the management involves revascularization and prevention of recurrence. We herein describe an anatomically annoying case of PSA aneurysm with recurrent embolism that necessitated thrombectomy and bypass surgery. And this work has been reported in line with the SCARE criteria.

**Case presentation:**

A 76-year-old woman developed leg embolism twice from PSA aneurysm. PSA was complete type and the popliteal artery ran on the lateral side, which was difficult to approach from the standard medial side. Based on these anatomical conditions, thrombectomy of the posterior-tibial artery (PTA) and femoral-PTA bypass were performed. Additionally, the proximal side of PTA from the anastomosis site was ligated to avoid the recurrence of embolism. Residual PSA aneurysm has not yet been treated, but neither clinical complications nor dilatation have occurred.

**Discussion:**

PSA is a rare disease, but should be remember in acute limb ischemia. Clear treatments have not yet been established; therefore, its plans need to be considered based on clinical symptoms and anatomical conditions.

**Conclusion:**

Although there were anatomical issues in the present case, thrombectomy and bypass surgery were successful and no complications have occurred one year after surgery.

## Highlights

• Recurrent embolism originated from the persistent persistent sciatic artery aneurysm.
• Distal bypass was required due to the anomaly position of popliteal artery.
• Persistent sciatic artery aneurysm is rare, but remember in especially acute limb ischemia.

## 1 Introduction

Persistent sciatic artery (PSA) is a rare congenital vascular anomaly. It has several clinical features due to the formation of aneurysms, leg ischemia, buttock pain or sciatic neuralgia [1,2] and aneurysm rupture [3]. The management of PSA causing leg ischemia involves revascularization and prevention of recurrence. We herein describe a case of PSA aneurysm with recurrent embolism that necessitated thrombectomy and bypass surgery. And this work has been reported in line with the SCARE criteria [4]

## 2 Case presentation

**Fig. 1:**
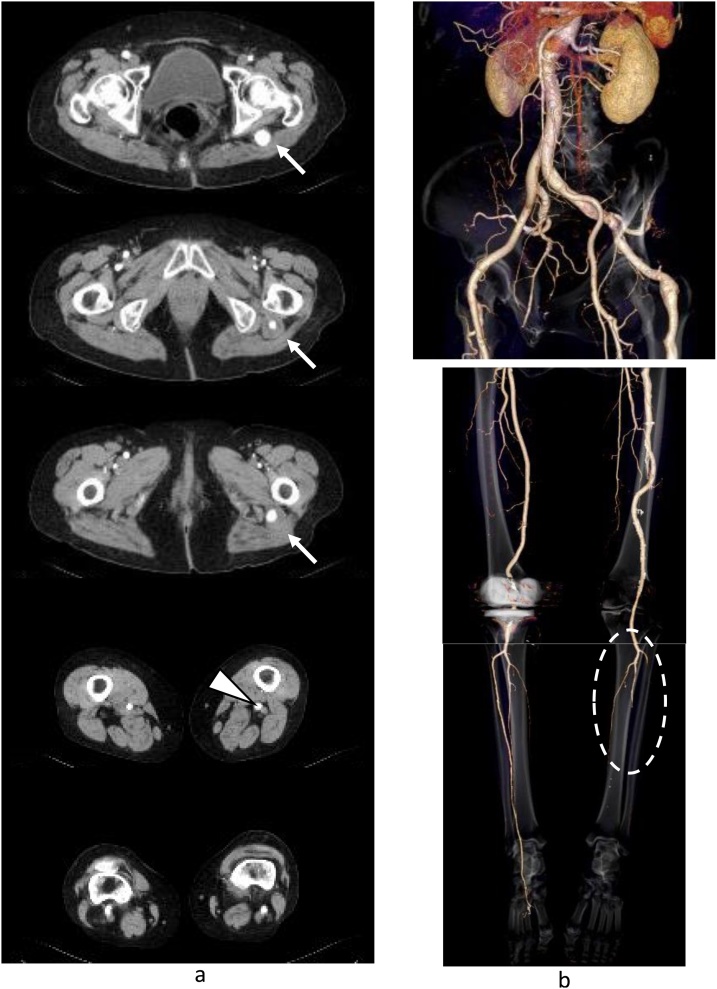
CT images at first admission. a. CT shows a left persistent sciatic artery (PSA) aneurysm (arrow) and the left popliteal artery located more laterally than the normal pattern (arrow head). b. Three-dimensional CT shows complete-type PSA, hypoplastic superficial femoral artery (7 mm), and occlusion in the three artery branches of the lower leg (circle).

**Fig. 2:**
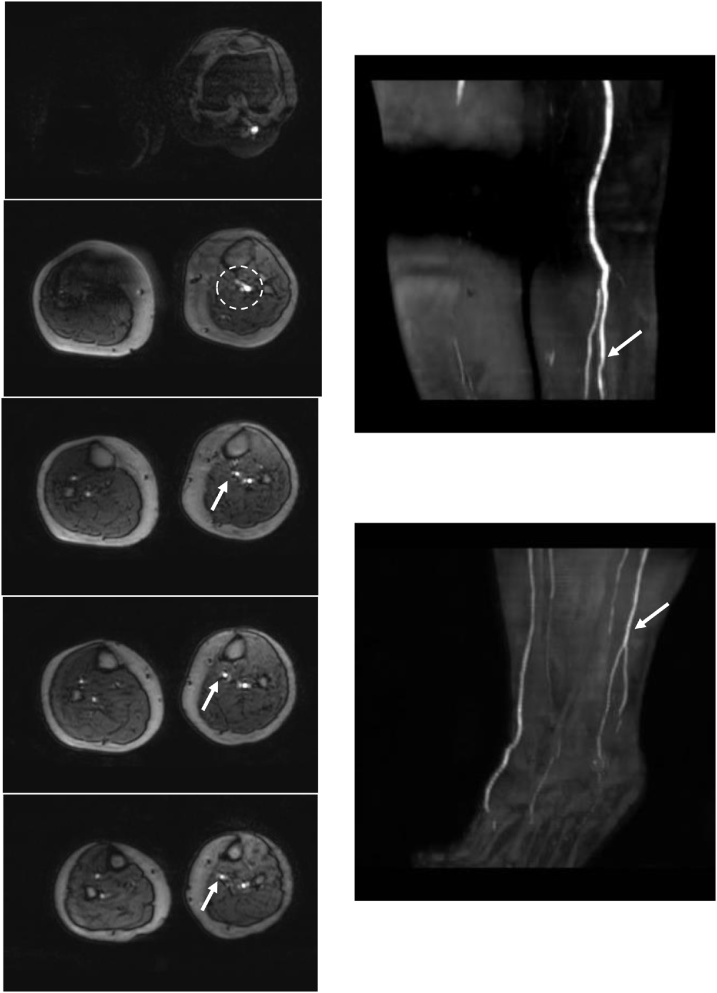
Magnetic resonance angiography during the first hospitalization. Magnetic resonance angiography shows the patency of the posterior-tibial artery (PTA) and peroneal artery (arrow) from the tibio-peroneal trunk (circle). The anterior-tibial artery was not detected.

**Fig. 3:**
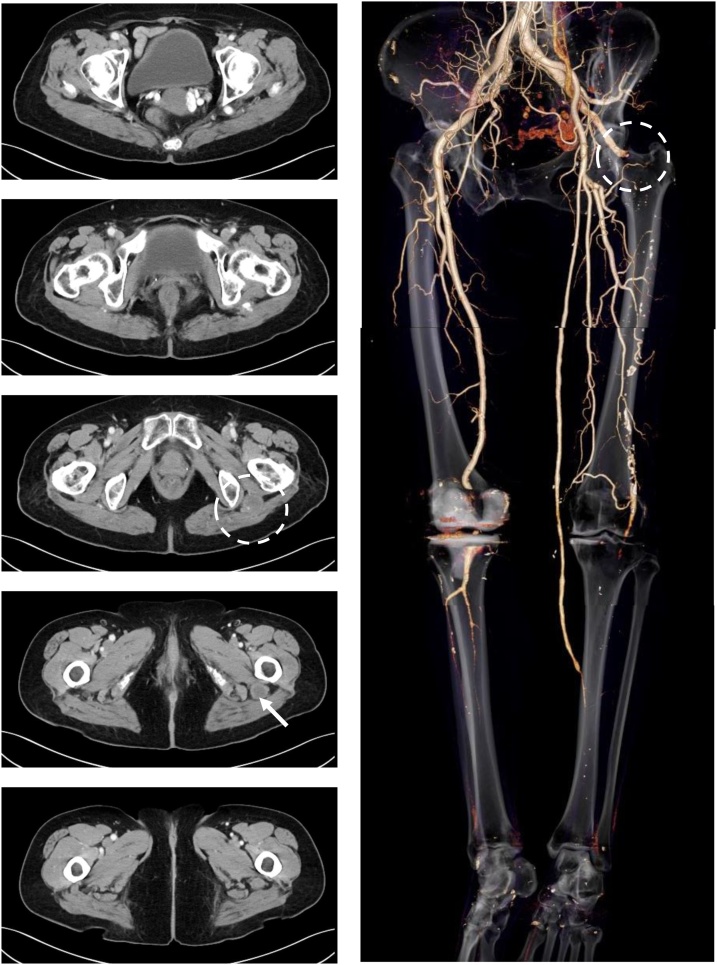
Enhanced CT images one year after the surgery. The bypass graft is patient and the artery from sciatic to superficial femoral is occluded at the PSA aneurysm (circle). The dilatation of PSA aneurysm has not been observed, its size is 22 mm in maximum minor-axis diameter (arrow).

A 76-year-old woman had been transferred to our hospital because the acute pain in the left lower leg. The lower extremity of affected side was cyanotic, while regular pulsation was palpable in the femoral artery (FA) and popliteal artery (PA). The previous medical history of this patient was hypertension and right total knee arthroplasty; however, arrhythmia had not been detected. CT scan revealed a left PSA aneurysm (Fig. 1a), the left PA fed from the PSA and the hypoplastic superficial FA (Fig. 1b). These results indicated “complete-type PSA” [1,5]. The PA ran more laterally than the normal pattern (Fig. 1a) and the three artery branches of the lower leg were occluded (Fig. 1b). The underlying cause was identified as embolic ischemia from the PSA aneurysm. Surgery was not performed because clinical symptoms rapidly improved with the administration of heparin. Conservative treatments by oral anti-platelet medicines and the dripped intravenous administration of heparin were continued for one week. MRI examination during hospitalization showed the patency of the posterior-tibial artery (PTA) and peroneal artery (Fig. 2). The patient was reluctant to surgical treatment to the PSA and discharged; however, the same symptoms recurred 6 months later and emergent surgical treatments were performed. Under general anesthesia, thrombectomy of PTA was performed and blood flow from distal side was observed. Next, the bypass between FA-PTA with a reversed saphenous vein graft were performed. Additionally, the proximal side of PTA from the anastomosis site was ligated to avoid the recurrence of embolism. One year after surgery, the recurrence of embolism and other complications have not occurred, furthermore the aneurysm is occluded by thrombus (Fig. 3).

## 3 Discussion

PSA has been estimated to occur in 0.01–0.06% of the population [2]. It has some clinical symptoms [[1], [2], [3]], as described in a review by Ikezawa et al. [1], these symptoms are related to the formation of PSA aneurysm which occurs in approximately 25–46% of cases [1,5]. Additionally, PSA is generally classified into two types: complete and incomplete. In complete type, PSA is continuous with PA directly and superficial FA is hypoplastic. Conversely, in incomplete type, PSA is hypoplastic and superficial FA is the major in-flow artery to the lower extremity [1,2]. A previous study reported that approximately 80% of PSA are the complete type [2], and the present case was complete type. These three factors: clinical features, aneurysm formation, and the PSA type influence the treatment plan [2]. A number of treatment methods have been employed, such as surgical resection or endovascular treatments (EVT) [2,6]. However, these remain controversial because of their associated complications. Injury to the sciatic nerve due to surgical resection has been reported, on the other hand, the long-term outcomes of EVT remain unknown [2].

In the present case, the clinical manifestation was ischemia, which is the most frequently reported complication [1]. Since there were no other complications, the aims of treatment were revascularization and prevention of the recurrence. Regarding the treatment plan, there were two anatomical issues: the hypoplastic superficial FA and the location of PA. In the former, the size of superficial FA was considered to be slightly small (diameter of 7 mm) to obtain sufficient bloodstream. In the latter, the standard FA-PA bypass was difficult, because the PA of the affected side ran more laterally than the normal pattern, which was difficult to approach from the conventional medial side. Therefore, three treatment plans were considered. #1: Direct resection and graft replacement of the aneurysm, #2: Bypass from the iliac artery to the PTA and ligation of the internal iliac artery at the proximal side of PSA and PTA, #3: The same method of present case. Regarding #1, if sciatic nerve injury occurred, gait disturbance may have become worse because this patient had previously undergone total knee arthroplasty on the opposite side. Concerning #2, this was considered to be too long to achieve long-term graft patency. Although method #3 was simple and less invasive, but the hypoplastic superficial FA was worried. However, considering the current condition of this patient, some treatments should be recommended before recurrence. The residual PSA aneurysm has not been treated because other complications have not occurred and the aneurysm is occluded by thrombus

## 4 Conclusion

We encountered a case of PSA aneurysm with recurrent embolism. Although PSA is rare, it should remember in the case of acute limb ischemia, and treatment plans need to be considered based on clinical symptoms and anatomical conditions.

## Declaration of Competing Interest

There are no conflicts of interest among all authors.

## Funding

This research did not receive any specific grant from funding agencies in the public, commercial, or not-for-profit sectors.

## Ethics approval

Not applicable for our case report.

## Consent

Written informed consent was obtained from the patient.

## Author contributions

Writing: Mutsuo Tanaka.

Critical review and revision: All authors.

Final approval of the article: All authors.

## Registration of research studies

None.

## Guarantor

All authors have read and approved and accept full responsibility for the work.

## Provenance and peer review

Not commissioned externally peer reviewed.
